# Giant Magnetic Purcell Effect Induced by High‐Order Mie Resonances

**DOI:** 10.1002/advs.76954

**Published:** 2026-08-07

**Authors:** Haonan Shi, Ningning Song, Anjun Huang, Zhengwen Yang, Jingbo Sun, Yongzheng Wen, Ji Zhou

**Affiliations:** ^1^ State Key Laboratory of New Ceramics and Fine Processing School of Materials Science and Engineering Tsinghua University Beijing China; ^2^ Faculty of Materials Science and Engineering Kunming University of Science and Technology Kunming China

**Keywords:** europium, magnetic purcell effect, mie resonance, titanium dioxide

## Abstract

The Purcell effect provides an effective route for tailoring light–matter interactions. However, efficient enhancement of magnetic dipole transitions remains challenging because magnetic light–matter coupling is intrinsically weak and strongly outcompeted by dominant electric dipole channels. Here we exploit high‐order Mie resonances supported by low‐loss TiO_2_ spheres to selectively enhance magnetic dipole emission from Eu^3+^ emitters. Numerical analysis shows that the coupling between a point emitter and Mie resonance increases with the resonance order, while the low optical loss of TiO_2_ enables near‐field Purcell enhancement to be converted into observable far‐field radiation. We further develop a surface‐grafting strategy that positions Eu^3+^ emitters within ∼2 nm of the sphere surface to maximize emitter–resonator coupling. Using single‐particle spectroscopy and a magnetic 32‐pole Mie resonance, we achieve a ∼8.7‐fold enhancement of the magnetic dipole transition branching ratio of Eu^3+^ ions, accompanied by an ∼38.1‐fold increase in the magnetic dipole decay rate. Our findings not only extend Mie‐tronics as a promising platform for manipulating light generation but also provide a versatile strategy for magnetic‐field‐driven nanophotonic functionalities.

## Graphical abstract

High‐order Mie resonances in dielectric spheres selectively amplify magnetic dipole emission from Eu^3+^ emitters, producing a giant magnetic Purcell effect. Localized emitters can efficiently access high‐order resonant modes, and the low‐loss TiO_2_ platform enables direct far‐field observation of the enhanced emission.

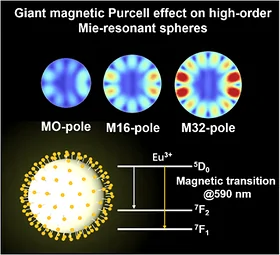

## 1 Introduction

The Purcell effect provides a fundamental framework for tailoring spontaneous emission through engineering of the photonic density of states [1, 2, 3]. In nanophotonics, developing structurally simple and efficient platforms for enhancing the Purcell effect has attracted sustained interest, such as nanoparticles‐on‐mirror [4] and nanogaps [5]. To date, substantial efforts have been devoted to enhancing electric dipole transitions, enabling efficient emission control in diverse systems, including single‐photon sources, rare‐earth ions, quantum dots, two‐dimensional materials, and molecular dyes [6, 7, 8, 9]. In contrast, the controlled enhancement of magnetic dipole transitions remains far less explored. This limitation arises from the intrinsically weak magnetic light–matter interaction, which is suppressed by the square of the fine‐structure constant (α ≈ 1/137) [10]. Addressing this limitation would unlock new opportunities in forbidden‐transition spectroscopy, macroscopic quantum phenomena, quantum information processing, and magnetic‐field‐based sensing, detecting, and imaging [11, 12, 13].

Several strategies have been proposed to enhance magnetic light–matter interactions through distinct design concepts. Near‐field scanning optical microscopy provides deeply subwavelength‐confined electromagnetic fields that enable access to magnetic transitions. Reynier et al., exploited this capability to selectively excite and map electric and magnetic dipole transitions in three dimensions [12]. Plasmonic nanostructures provide another route by generating magnetic hotspots. Choi et al., employed plasmonic Au nanocavity arrays to selectively enhance magnetic‐dipole emission in Er^3+^ ions, achieving up to a 26‐fold enhancement in fluorescence intensity [14]. Liang et al., further utilized vortexed plasmonic nanocavities to synergistically enhance magnetic‐dipole transitions and circularly polarized luminescence in Eu^3+^ complexes, opening opportunities for displays and quantum photonic applications [15]. Dielectric metasurfaces offer an alternative approach through engineered magnetic photonic density of states [16, 17, 18, 19, 20]. Vaskin et al., demonstrated preferential enhancement of magnetic‐dipole emission in Eu^3+^ ions using Si‐based Mie‐resonant metasurfaces, enabling control over the electric–magnetic emission branching ratio [21]. High‐quality factor (*Q*) resonances provide a further degree of enhancement [22]. Bashiri et al., utilized *quasi*–bound states in the continuum mode with high‐*Q* factor in a TiO_2_ metasurface to achieve a ∼15‐fold enhancement of the magnetic‐to‐electric dipole transition branching ratio of Eu^3+^ ions [23]. Beyond structural engineering, magnetic transitions can also be selectively addressed using structured light fields. Montagnac et al., showed selective excitation of electric‐ or magnetic‐ dipole transitions in rare‐earth ions using tightly focused cylindrical vector beams with spatially separated electric and magnetic field components [24]. Despite these advances, such strategies require complex fabrication or sophisticated excitation schemes.

Dielectric Mie‐resonant spheres provide an attractive alternative. Although they do not possess the extreme electric field confinement of metallic counterparts, their intrinsically low optical losses and strong magnetic responses arising from circulating displacement currents provide a natural platform for enhancing magnetic light–matter interactions [25, 26, 27, 28]. In a pioneering study, Sugimoto et al., achieved a ∼7‐fold enhancement in the magnetic‐to‐electric dipole transition branching ratio of Eu^3+^ ions by coupling emitters to the magnetic quadrupole (MQ) mode of silicon Mie‐resonant spheres [27]. In their subsequent work, the magnetic octupole (MO) mode was explored to further improve the performance of silicon spheres [29]. ZrO_2_ spheres and Au‐ZrO_2_ sphere dimers have also been explored to tailor magnetic emission channels, yet the reported branching ratio enhancement remains below twofold [30]. However, to date, these efforts have been limited to low‐order Mie resonances. Higher‐order modes offer larger *Q*‐factors and thus greater potential for enhancing the Purcell effect, but they are conventionally considered difficult to excite and remain unexploited.

Here, we demonstrate that low‐loss TiO_2_ spheres supporting high‐order Mie resonances provide an effective platform for tailoring the magnetic Purcell effect. By anchoring a monolayer of Eu^3+^ complexes within 2 nm of the sphere surface, we enable the excitation of high‐order modes and achieve efficient coupling between Eu^3+^ magnetic dipoles and spectrally aligned resonances. Utilizing high‐order Mie resonances leads to pronounced enhancement of both the magnetic dipole transition efficiency and the corresponding radiative decay rate. These results not only demonstrate high‐order Mie resonances as powerful tools for controlling light generation, but also highlight simple dielectric spheres as versatile systems for engineering magnetic light–matter interactions at the nanoscale.

## 2 Results and Discussion

Figure 1 presents the simulation results of Mie‐resonant spheres for enhancing electronic transitions. Figure 1a shows the scattering efficiency spectrum and corresponding multipole decomposition of a 500 nm TiO_2_ sphere under plane‐wave excitation. The scattering efficiency is defined as Equation (1) [31].

(1) $$Q_{scat}=\frac{P_{scat}}{P_{inc}}$$

where, *P*_scat_ and *P*_inc_ are scattered and incident (plane wave or dipoles) radiation powers, respectively. The scattering response can be decomposed into multipolar contributions of different orders, which can be further classified into angular and radial modes. The angular modes are denoted as electric dipole(ED)/magnetic dipole(MD), electric quadrupole(EQ)/MQ, and higher‐order multipoles, whereas the radial modes are denoted as *n*‐th‐order resonances within each angular momentum channel (i.e., *n*‐th‐order ED/MD, EQ/MQ, etc.). To better visualize the high‐order spectral region, Figure 1b presents an enlarged view of the scattering efficiency spectrum. It can be seen that both high‐angular‐order and high‐radial‐order modes coexist within this wavelength range. Figure 1c compares the scattering spectra of TiO_2_ spheres under plane‐wave excitation and excitation by 100 randomly distributed dipoles located 2 nm above the sphere surface. Pronounced differences are observed between the two excitation schemes. Under plane‐wave excitation, the scattering spectrum contains contributions from both angular and radial modes, and the excitation efficiency of high‐angular‐order modes generally decreases with increasing mode order. In contrast, dipole excitation preferentially excites higher‐angular‐order modes, while the excitation of higher‐radial‐order modes is suppressed, leading to effective mode separation. This excitation selectivity originates from the stronger spatial overlap between the localized near field of the dipole emitters and the field distributions of high‐angular‐order modes. As a result, surface emitters can efficiently access resonances that are only weakly excited by plane waves, providing a favorable route for enhancing light–matter interactions.

**FIGURE 1:**
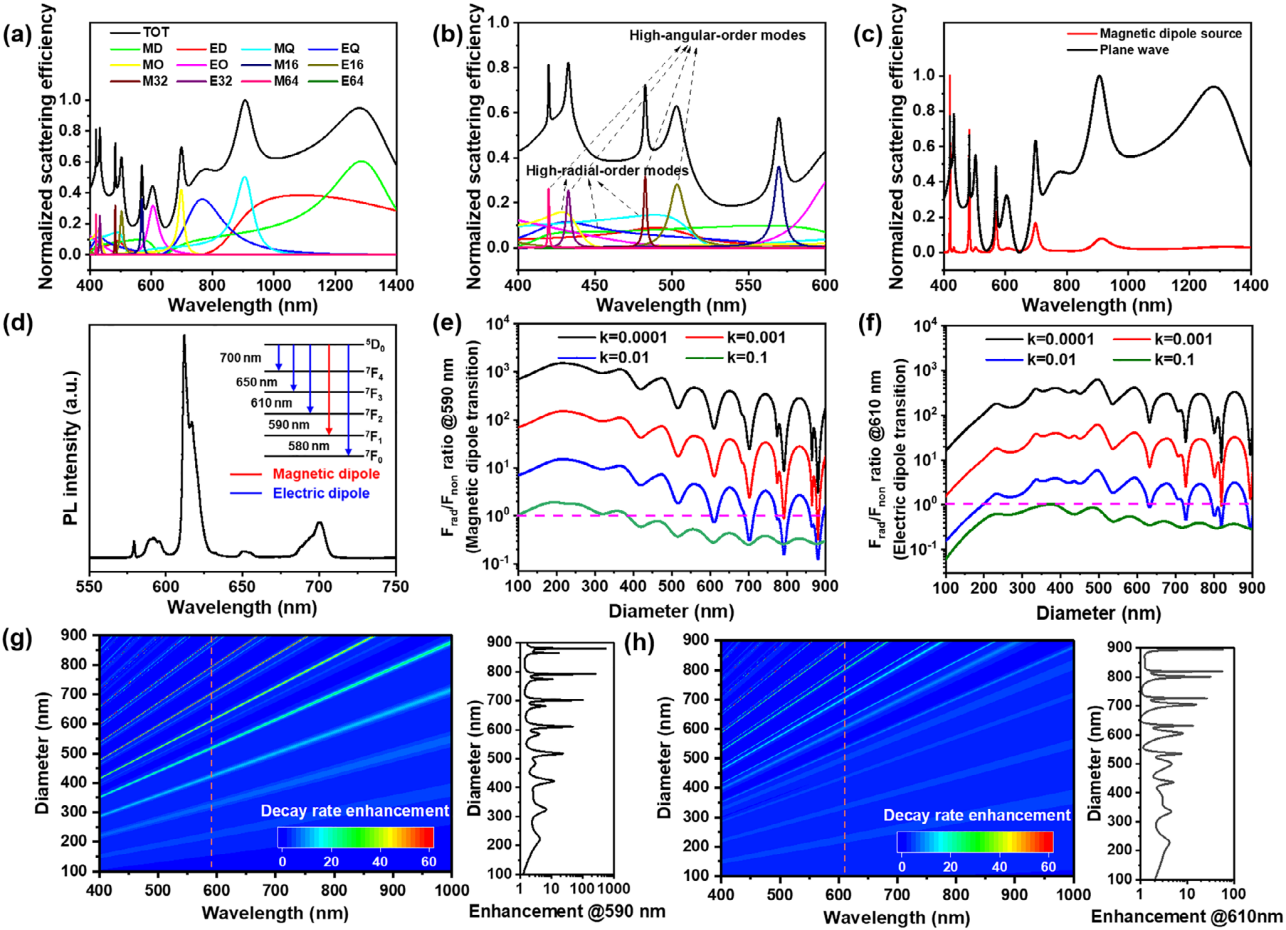
Simulation of Mie‐resonant spheres for electron transition enhancement. (a) Scattering efficiency spectrum and corresponding multipole decomposition of a 500 nm TiO_2_ sphere under plane wave excitation; (b) Magnified view of the high‐order‐mode region of the scattering spectrum; (c) Comparison of the scattering spectra under plane‐wave excitation and magnetic‐dipole‐emitter excitation (100 dipoles with random distribution and orientation); (d) 4f energy level diagram of Eu^3+^ ion; (e, f) Effect of loss on the radiative‐to‐nonradiative enhancement ratio of (e) an isotropic magnetic dipole and (f) an isotropic electric dipole; (g, h) Radiative rate enhancement factors for (g) an isotropic magnetic dipole and (h) an isotropic electric dipole.

To experimentally verify the enhancement of magnetic light–matter interactions enabled by high‐order Mie resonances, Eu^3+^ ions were chosen as a model emitter owing to their well‐defined electric‐ and magnetic‐dipole transitions originating from the same excited state. Figure 1d shows the energy‐level diagram of Eu^3+^ ions. The ^5^D_0_→^7^F_1_ transition at ∼590 nm corresponds to a magnetic dipole transition, whereas the ^5^D_0_→^7^F*_j_* (*j* = 0,2–4) transitions at ∼580, ∼610, ∼650, and ∼700 nm are electric dipole transitions. The diameters of the TiO_2_ spheres were tuned so that their Mie‐resonant frequencies match the targeted electron transitions, enabling selective modulation of the corresponding emission channels.

Under the weak‐coupling regime, we simulated the radiative rate enhancement factors of TiO_2_ spheres for different Eu^3+^ transitions. Figure 1g,h shows the enhancement factors for isotropic electric and magnetic dipole emitters as functions of sphere diameter and wavelength. Magnetic dipole transitions experience a stronger enhancement than the electric dipole transition, highlighting the advantage of dielectric Mie resonators in mediating magnetic light–matter interactions. In addition, magnetic‐type resonant modes provide stronger enhancement for both electric and magnetic emitters located near the sphere surface than electric‐type modes. This behavior arises because the displacement currents associated with magnetic resonant modes are concentrated closer to the sphere surface than those of electric modes (Figure S1). For magnetic dipole transitions, a dipole oriented perpendicular to the sphere surface exhibits higher enhancement than a parallel dipole (Figure S2). Table 1 summarizes the calculated cavity parameters of TiO_2_ Mie‐resonant spheres. Since this work focuses on magnetic light–matter interactions, the conventional mode volume definition based on electric field enhancement is not directly relevant to the magnetic properties of the system and therefore cannot adequately characterize magnetic light–matter coupling. Therefore, we introduce a magnetic mode volume to describe the effective confinement of magnetic fields. In analogy with the electric Purcell effect, the magnetic effective mode volume is defined in Equation (2).

(2) $$V_{\mathrm{eff}}-H=\frac{\int_{V}\mu \left(\vec{r}\right)|\vec{H}\left(\vec{r}\right){|}^{2}dV}{\max\left[\mu \left(\vec{r}\right)|\vec{H}\left(\vec{r}\right){|}^{2}\right]}$$

**TABLE 1:** Calculated cavity parameters of TiO_2_ Mie‐resonant spheres.

Mode order	MQ	MO	M16‐pole	M32‐pole	M64‐pole
Radius (nm)	163.1	211.4	259.9	305.6	351.3
*Q*‐factor	13	32	79	205	542
*V*_eff_ ‐H((*λ*/*n*)^3^)	0.3	0.4	0.6	0.7	0.9
Purcell factor	6.7	11.4	17.3	48.5	120.4

As the resonance order increases, the *Q*‐factor rises from 13 to 545, whereas the effective mode volume increases only slightly and remains on the order of ∼(*λ*/*n*)^3^. This scaling makes higher‐order Mie resonances particularly favorable for achieving strong magnetic Purcell enhancement. The theoretical magnetic Purcell factors corresponding to the MQ, MO, M16‐pole, M32‐pole, and M64‐pole are 6.7, 11.4, 17.3, 48.5, and 120.4, respectively. The Purcell factors obtained from finite‐element simulations (COMSOL Multiphysics) are identical to the radiative‐rate enhancement factors derived using Schmidt's formulation [32].

Another key factor that governs the observable emission enhancement is material loss. Although high‐order Mie resonances can exhibit strong Purcell enhancement, optical absorption introduces competing nonradiative decay channels that reduce the far‐field radiative emission. Figure 1e,f and Figure S3 illustrate the influence of material loss on the ratio between radiative and nonradiative enhancement. As the loss increases, the radiative‐to‐nonradiative enhancement ratio decreases markedly. Moreover, higher‐order Mie resonances exhibit a larger proportion of nonradiative enhancement, indicating that they are more sensitive to material absorption.

To further clarify the role of material properties and the advantages of TiO_2_ spheres, we simulated the emission enhancement induced by Mie resonances in Si and Au spheres (Figure S4). For Si spheres, when the resonance order exceeds the MO mode, the nonradiative enhancement becomes larger than the radiative enhancement, consistent with previous reports by Sugimoto [27, 29]. Furthermore, Au spheres cannot efficiently support displacement‐current‐driven magnetic resonances, leading to a very limited enhancement of magnetic dipole transitions (<10). The intrinsic Ohmic loss of Au also leads to radiative enhancement that is consistently weaker than the corresponding nonradiative enhancement. These results highlight the advantage of low‐loss TiO_2_ spheres supporting high‐order Mie resonances for enhancing magnetic light–matter interactions.

The above theoretical analysis indicates that strong magnetic‐dipole emission enhancement requires the simultaneous fulfillment of three conditions: (i) excitation of high‐order Mie resonances with large Purcell factors, (ii) suppression of nonradiative losses through the use of low‐loss dielectric materials, and (iii) efficient near‐field coupling between emitters and resonant modes. To experimentally realize these conditions, TiO_2_ spheres were selected as the resonant platform owing to their low optical loss and ability to support high‐order magnetic Mie resonances. Eu^3+^ ions were then positioned close to the sphere surface to maximize emitter–resonator coupling. We next examined the influence of the distance between Eu^3+^ emitters and the TiO_2_ sphere surface on magnetic dipole emission enhancement. Calculations show that the enhancement decreases as the separation increases from 1 to 10 nm (Figure S5), suggesting the importance of positioning the emitters within the near‐field region of the Mie resonances. To minimize this distance, short‐chain 2,2'‐bipyridine‐4,4'‐dicarboxylic (bpdc) ligands were employed to anchor Eu^3+^ ions close to the TiO_2_ surface. Figure 2a shows the synthesis route of Eu^3+^‐grafted TiO_2_ spheres (TiO_2_‐OH@Eu(TTA)_3_bpdc) via a surface ligand‐exchange strategy. The size of the TiO_2_ spheres was modulated by varying the synthesis temperature (0, 20, and 40°C), with the diameter decreasing as the temperature increased (Figure S6). The TiO_2_ spheres were first functionalized with bpdc ligands, followed by coordination with Eu(TTA)_3_ complexes. SEM and TEM images (Figure 2b) reveal highly spherical TiO_2_ spheres with good crystallinity. HRTEM images and corresponding SAED patterns (Figure 2b) confirm a polycrystalline anatase structure, consistent with the XRD results (Figure 2c). No detectable phase evolution is observed after Eu^3+^ grafting (Figure 2c). TEM–EDS mapping and line‐scanning analyses show a homogeneous Eu‐containing shell on the TiO_2_ surface with a thickness below 2 nm. SEM‐EDS mapping further confirms uniform Eu coverage across all spheres rather than individual spheres (Figure S7). IR spectra (Figure 2e) verify the coordination of Eu^3+^ ions. The disappearance of the ─COOH vibration band indicates coordination between surface carboxylate groups and Eu^3+^ ions, while redshifts of the C═N and C─O vibrations relative to Eu(TTA)_3_(H_2_O)_2_ confirm ligand exchange and successful anchoring of the Eu^3+^ complex [33, 34]. As a reference sample, P‐TiO_2_@Eu(TTA)_3_bpdc was prepared using commercially available anatase TiO_2_ powder. Its morphology (Figure S8) exhibits irregular aggregated structures, in contrast to the well‐defined spherical morphology of the synthesized TiO_2_ particles.

**FIGURE 2:**
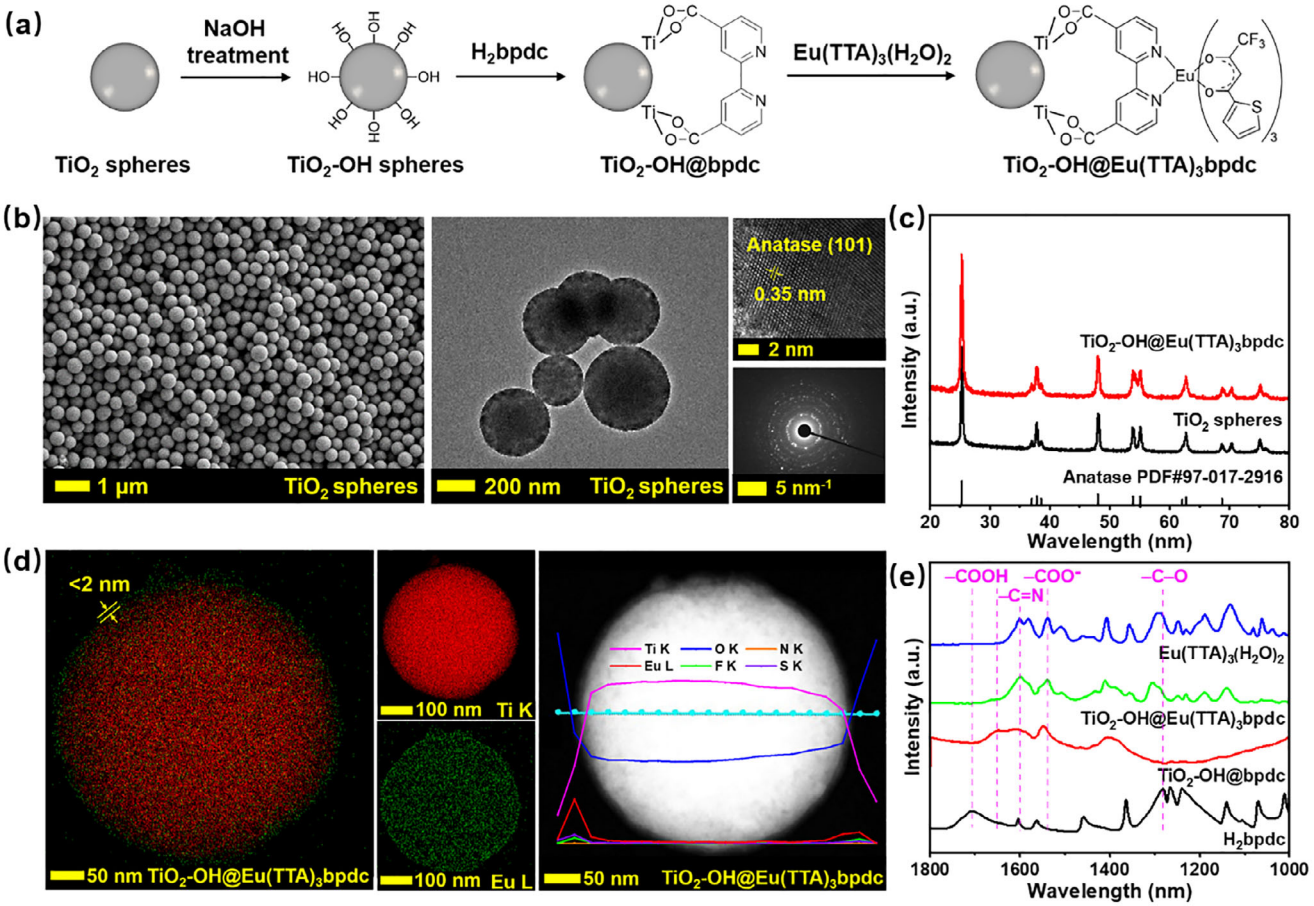
Synthesis and structure of TiO_2_ and Eu^3+^‐grafted TiO_2_ spheres. (a) Schematic of synthesis route; (b) SEM image, TEM image, HRTEM image, and corresponding SAED pattern of TiO_2_ spheres; (c) XRD patterns of TiO_2_ and Eu^3+^‐grafted TiO_2_ spheres; (d) TEM‐EDS mapping and line‑scanning of Eu^3+^‐grafted TiO_2_ spheres; (e) FTIR spectra.

Figure 3 presents the steady‐state emission enhancement mediated by Mie resonances. To enable accurate particle localization, the TiO_2_ spheres were dispersed on a glass substrate with photolithographically patterned markers (Figure S9). Dark‐field scattering images reveal distinct colors for spheres with different diameters, reflecting their different Mie‐resonant characteristics (Figure S10). To quantitatively evaluate the modulation of Eu^3+^ emission, the electric dipole transitions (^5^D_0_→^7^F*_j_*, *j* = 0, 2–4) were used as an internal reference. This approach minimizes the influence of particle‐to‐particle variations in the amount of grafted Eu^3+^ complexes and enables a more reliable comparison between different resonant spheres. The magnetic‐transition branching ratio is calculated according to Equation (3) [27].

(3) $$\beta_{j}^{\mathrm{cal}}=\frac{\Gamma_{j}\beta_{j}^{\mathrm{ref}}}{\sum_{j=0}^{4}\Gamma_{j}\beta_{j}^{\mathrm{ref}}}$$

where $\beta_{j}^{ref}$ is the branching ratio of the reference P‐TiO_2_–OH@Eu(TTA)_3_bpdc, *Γ_j_* is the calculated radiative rate enhancement factors of ^5^D_0_ →^7^F*_j_* (*j* = 0–4) transitions. The theoretical branching ratios for different transitions are shown in Figure S11, indicating that high‐order Mie‐resonate modes can selectively and efficiently enhance specific Eu^3+^ transitions depending on their spectral overlap with the corresponding resonances. The measured branching ratio is defined as Equation (4) [27].

(4) $$\beta_{j}^{\exp}=\frac{I_{j}}{\sum_{j=0}^{4}I_{j}}$$

where *I_j_* is the photoluminescent intensity of a ^5^D_0_ →^7^F*_j_* transition.

**FIGURE 3:**
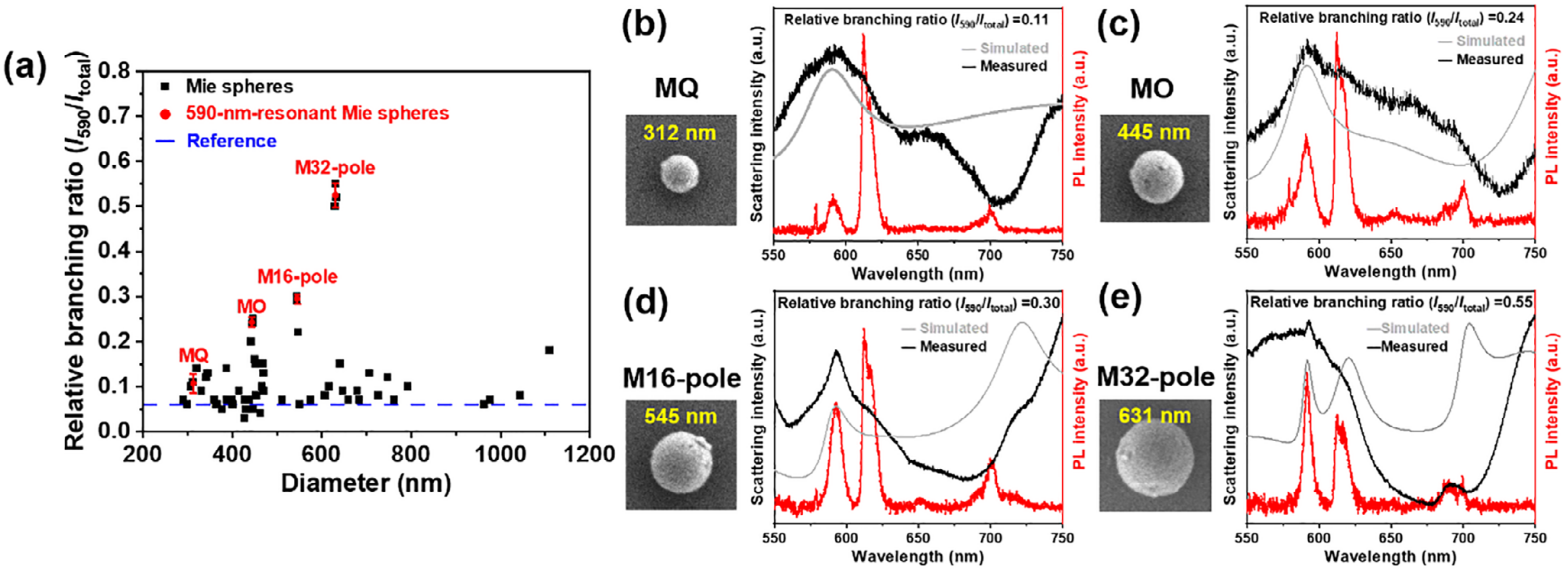
Steady‐state emission enhancement mediated by Mie resonances. (a) Magnetic dipole transition branching ratio (I_590_/I_total_) of TiO_2_–OH@Eu(TTA)_3_bpdc and the reference P‐TiO_2_–OH@Eu(TTA)_3_bpdc; (b–e) Measured (black curves) and simulated (gray curves) scattering spectra, and photoluminescence spectra (red curves) of representative TiO_2_@Eu(TTA)_3_bpdc corresponding to (b) MQ, (c) MO, (d) M16‐pole, and (e) M32‐pole resonant modes.

The photoluminescence spectrum of the reference sample is shown in Figure S12. The emission is dominated by the electric‐dipole transition at ∼610 nm, while the magnetic‐dipole transition at ∼590 nm exhibits a branching ratio of 0.06. The measured magnetic‐dipole branching ratios are summarized in Figure 3a. Pronounced enhancement is observed only at sphere diameters of approximately 312, 445, 545, and 631 nm, whereas the branching ratio remains close to that of the reference sample at other diameters. These characteristic diameters correspond to the excitation of different Mie‐resonant modes spectrally matched to the Eu^3+^ magnetic‐dipole transition.

Figure 3b–e shows the measured and simulated scattering spectra, together with the corresponding photoluminescence spectra, for TiO_2_ spheres supporting the MQ, MO, M16‐pole, and M32‐pole resonances. The black curves represent the experimentally measured scattering spectra, while the gray curves correspond to simulations performed with a SiO_2_ substrate (*n* = 1.45). During the sintering process, size‐dependent porosity may reduce the effective refractive index of the spheres [35]. By matching the simulated and experimental spectra, the effective refractive indices at ∼590 nm are estimated to be 2.55 (∼312 nm), 2.32 (∼445 nm), 2.32 (∼545 nm), and 2.35 (∼631 nm), respectively. To further verify the mode assignment, multipole decomposition analyses were performed and are presented in Figure S13. The results confirm that the observed resonances correspond to the MQ, MO, M16‐pole, and M32‐pole modes, respectively. The measured magnetic‐dipole branching ratios for these modes are 0.11 ± 0.02, 0.24 ± 0.01, 0.30 ± 0.01, and 0.52 ± 0.03, corresponding to 1.8‐, 4.0‐, 5.0‐, and 8.7‐fold enhancements relative to the reference sample.

Enhancement can also occur when other emission transitions spectrally align with Mie resonances. For example, the electric dipole emission near 580 nm is enhanced when it matches the M32‐pole resonance, reaching a branching ratio of ∼0.4, corresponding to an enhancement of ∼7.4‐fold (Figure S14). Although numerical simulations suggest that even stronger enhancement may be achievable with higher‐order resonances, experimental investigation beyond the M32‐pole mode was not pursued. This is because higher‐order modes exhibit extremely weak dark‐field scattering signals and increasingly narrow linewidths, making their experimental identification and spectral matching substantially more challenging.

Figure 4 presents the time‐resolved photoluminescence enhancement mediated by TiO_2_ Mie‐resonant spheres. A confocal time‐resolved fluorescence microscope was used to measure the radiative lifetimes of individual particles at 590 nm. The decay curves are fitted by a single‐exponential model, *I*(*t*)/*I*_0_(*t*) = *A*exp(‐*t*/*τ*)+*y*_0_. The lifetime of the reference sample P‐TiO_2_–OH@Eu(TTA)_3_bpdc is 215.5 ± 0.8 µs (Figure S15). To investigate the influence of the M32‐pole resonance, we measured both resonant TiO_2_ spheres and nonresonant sphere aggregates. The measurement region is shown in Figure 4a, and the corresponding lifetime mapping is presented in Figure 4b. Lifetimes of ∼200 µs are observed across most regions, consistent with the reference sample. In contrast, the lifetime is markedly shortened at the M32‐pole position. The normalized decay curves measured at the M32‐pole position and at three nonresonant locations (P1–P3) are presented in Figure 4c. The extracted lifetime for M32‐pole Mie‐resonant sphere is 51.8 ± 0.9 µs, while the lifetimes of P1, P2, and P3 are 202.1 ± 1.8 µs, 181.8 ± 1.2 µs, and 207.5 ± 1.1 µs, respectively. These results indicate that the emission dynamics remain largely unchanged away from resonance, whereas coupling to the M32‐pole mode leads to a pronounced acceleration of the decay process.

**FIGURE 4:**
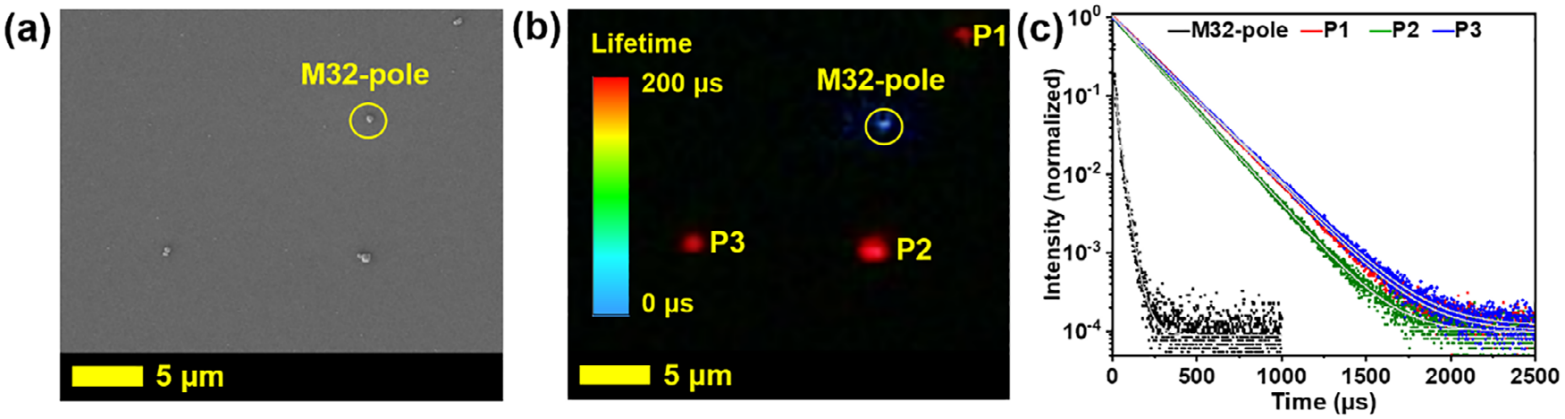
Time‐resolved fluorescence at 590 nm mediated by Mie resonances. (a) Spatial positioning of TiO_2_ spheres supporting M32‑pole Mie‐resonant modes; (b) Photoluminescence lifetime mapping; (c) Normalized time‑resolved decay curves.

Because the monitored 590 nm emission originates from the ^5^D_0_ energy level, the measured lifetime reflects the total decay rate of this level, including contributions from all radiative transitions ^5^D_0_→^7^F_n_ (*n* = 0–5). Therefore, the enhancement of the magnetic‐dipole transition rate at 590 nm cannot be obtained directly from the lifetime reduction alone and requires correction using the experimentally measured branching ratios. According to Equation (5), the enhancement factor at 590 nm can be obtained from the measured total radiative rate enhancement factor *G* and the branching ratio *β*.

(5) $$\frac{\Gamma_{590,enhanced}}{\Gamma_{590,ref}}=\frac{\beta_{590,enhanced}}{\beta_{590,ref}}\times G_{\mathrm{total},\mathrm{enhanced}}$$

Using this correction, the enhancement factor of the 590 nm magnetic‐dipole transition is determined to be ∼38.1. This value is lower than the theoretical Purcell factor (∼48.5) listed in Table 1. This can be attributed to several factors, including substrate‐induced perturbations, refractive‐index dispersion of TiO_2_, residual optical losses, and deviations from the ideal spatial and orientational overlap between the emitters and the Mie‐resonant mode.

Table 2 compares the performance of the present work with relevant literature. Most previous studies quantify the enhancement of magnetic dipole emission of Eu^3+^ ions using the intensity ratio *I*_590_/*I*_610_. However, because additional emission channels also contribute to the total radiative output, we adopt the ratio *I*_590_/*I*_total_ (*I*_total_ = *I*_580_+*I*_590_+*I*_610_+*I*_650_+*I*_700_) to provide a more consistent comparison across different systems. As summarized in Table 2, the present TiO_2_ Mie‐resonant platform delivers state‐of‐the‐art performance in magnetic‐dipole branching‐ratio enhancement. These results demonstrate the effectiveness of high‐order Mie resonances for manipulating magnetic light–matter interactions and highlight low‐loss TiO_2_ resonators as a promising platform for magnetic‐field‐driven nanophotonic functionalities.

**TABLE 2:** Comparison of magnetic dipole emission enhancement of Eu^3+^ ions with literature.

No.	Optical structure	Enhancement of *I*_590_/*I*_610_	Enhancement of *I*_590_/*I*_total_
1	Si‐based metasurface [21]	∼1.7‐fold	—
2	Cylindrical Si antenna [36]	∼2‐fold	—
3	Magnetic quadrupole mode of Si spheres [27]	∼11‐fold	∼7‐fold
4	Magnetic octupoles mode of Si spheres [29]	∼5‐fold	∼4‐fold
5	Magnetic dipole mode of ZrO_2_ spheres [30]	∼1.5‐fold	∼1.3‐fold
6	Au–ZrO_2_ sphere dimers [30]	∼1.3‐fold	∼1.2‐fold
7	*quasi*‐BIC mode of TiO_2_ metasurface [23]	∼15‐fold	∼7‐fold
8	Metallic antenna excited by azimuthally polarized beam [37]	∼4.5‐fold (excitation)	—
9	Magnetic 32‐pole mode of TiO_2_ spheres (This work)	∼20.8‐fold	∼8.7‐fold

## 3 Conclusion

In this work, low‐loss TiO_2_ spheres supporting high‐order Mie resonances are demonstrated as a structurally simple and effective platform for tailoring magnetic light–matter interactions. The coupling between localized emitters and high‐order Mie modes leads to pronounced magnetic Purcell enhancement, while the low optical loss of TiO_2_ allows the enhanced emission to be converted into observable far‐field radiation. By employing a surface‐grafting strategy, Eu^3+^ emitters are positioned uniformly within ∼2 nm of the sphere surface to maximize emitter–resonator coupling. Using single‐particle measurements, we experimentally observe a ∼8.7‐fold enhancement of the magnetic dipole emission branching ratio and a ∼38.1‐fold increase in the radiative decay rate. These results highlight the TiO_2_ dielectric spheres as a promising platform for engineering magnetic light–matter interactions and advancing magnetic photonic functionalities.

## 4 Experimental Section

### 4.1 Electromagnetic Simulation

The refractive index of the TiO_2_ spheres (anatase phase) was set as 2.45 [38, 39]. The scattering spectra, magnetic field distribution, *Q*‐factor, and effective mode volume of individual TiO_2_ spheres were simulated using the wave optics module in COMSOL Multiphysics, with perfectly matched layers (PML) surrounding the structure. Mie multipole theory is used to describe the sphere's resonant response [40]. The decay rates of electric and magnetic dipole sources near TiO_2_ spheres were calculated based on Schmidt's formulation [32]. The total enhancement for an isotropic emitter is obtained by averaging the decay rates of dipoles oriented parallel and perpendicular to the sphere surface. For the Purcell factor calculation, the emitter was modeled as a magnetic dipole located 2 nm away from the sphere surface, corresponding to the average thickness of the Eu^3+^ coordination layer determined from TEM characterization. A virtual spherical surface with a diameter of 4 nm surrounding the emitter was introduced to calculate the emitted power. The total emitted power was obtained by integrating the Poynting vector over this surface, *P*_tot_ = ∯ ***S*** ·d ***A***. The magnetic Purcell factor is given by *F*_M_  =  *P*_tot_/*P*_M_, where *P*_M_ is the power radiated by the corresponding dipole in free space. The final Purcell factor is obtained by averaging the Purcell factors for dipoles oriented parallel and perpendicular to the sphere surface.

### 4.2 Materials

Acetonitrile (GC, >99.5%), methanol (AR, 99.5%), ethanol (AR, 99%), N,N‐Dimethylformamide (DMF) (GC, 99.9%), titanium isopropoxide (TTIP) (AR, >98%), EuCl_3_·6H_2_O(AR, 99.99%), dodecylamine (AR, 98%), 2,2'‐Bipyridine‐4,4'‐dicarboxylic acid (H_2_bpdc) (AR, 96%), NaOH (AR, >96%), and titanium oxide (anatase, 99.9%) was purchased from Macklin Ltd. Thenoyltrifluoroacetone (AR, 98%) was purchased from Konoscience Ltd. All materials were used as received without further purification.

### 4.3 Preparation of TiO_2_ Spheres

TiO_2_ spheres were synthesized using a sol–gel method. In a typical procedure, dodecylamine (0.5669 g, 2 mmol) and deionized water were dissolved in a mixed solvent of methanol and acetonitrile (35:15 mL, V/V). After stirring for 30 min at a specific temperature, titanium isopropoxide (TTIP) was added dropwise. The diameter of TiO_2_ spheres can be tailored by varying H_2_O/TTIP ratio and temperature (Figure S1). The mixture was then stirred and aged for 8 h. The resulting precipitate was collected by centrifugation, washed three times with methanol, and dried overnight. Finally, the product was calcined in air at 500°C for 4 h.

### 4.4 Preparation of Eu(TTA)_3_bpdc‐Grafted TiO_2_ Spheres and Bulk Particles

First, TiO_2_ spheres were hydroxyl‐functionalized in 5 m NaOH for 24 h. The solids were then washed with 0.1 m HCl and deionized water until the filtrate pH ≈ 6. The product was dried at 60°C in vacuum for 12 h, which is presented as TiO_2_–OH. Second, 0.05 g TiO_2_–OH and 0.10 g H_2_bpdc were dispersed in 20 mL DMF under ultrasonication. The mixture was refluxed at 160°C for 6 h under stirring. The product was then collected by centrifugation and washed with DMF at 80°C and ethanol at room temperature. After that, the sample was dried at room temperature for 12 h, which is presented as TiO_2_–OH@bpdc. Finally, 0.10 g TiO_2_–OH@bpdc and 0.5 mmol Eu(TTA)_3_(H_2_O)_2_ were dispersed in 20 mL ethanol under ultrasonication. Eu(TTA)_3_(H_2_O)_2_ was synthesized following a reported procedure [41]. The mixture was then refluxed at 80°C for 5 h. The product was collected by centrifugation and washed with ethanol. After that, the sample was dried at room temperature for 12 h, which is presented as TiO_2_–OH@Eu(TTA)_3_bpdc. Eu(TTA)_3_bpdc‐grafted TiO_2_ nonspherical particles were synthesized in a similar approach but using commercially available anatase TiO_2_ particles as the starting material, which are presented as P‐TiO_2_–OH@Eu(TTA)_3_bpdc.

### 4.5 Material Characterization

Scanning electron microscopy (SEM) was carried out on a Zeiss Merlin microscope (Carl Zeiss AG, Germany) at an accelerating voltage of 5 kV. Transmission electron microscopy (TEM) was carried out on a JEM‐F200 microscope (JEOL Ltd., Japan) at an accelerating voltage of 200 kV. X‐ray diffraction (XRD) patterns were obtained using a Bruker D8 diffractometer (Bruker Corporation, Germany). Fourier‐transform infrared (FTIR) spectroscopy was performed on a Nicolet iS50 spectrometer (Thermo Fisher Scientific, USA) equipped with an attenuated total reflectance (ATR) accessory.

### 4.6 Optical Spectroscopy

For single‐particle characterization, the samples were dispersed onto a glass substrate (thickness 0.21 mm) patterned with coordinate markers fabricated by photolithography. A Ti adhesion layer followed by an Au marker layer was sequentially deposited to define the reference array. Single‐particle dark‐field scattering spectra were recorded using a Nikon Ti‐2 inverted optical microscope. The substrate was placed face‐down on the microscope stage. The sample was illuminated using a halogen lamp coupled to a dry dark‐field condenser (NA = 0.95–0.80, Nikon). The scattered light from individual particles was collected by a 50× objective lens (NA = 0.6, Nikon) and directed to the entrance slit of an HiperS‐3328iMS‐TH imaging spectrometer (Zolix, China) for spectral acquisition. Photoluminescent measurements were performed on an alpha 300R confocal imaging system (WITec, Germany). The sample was excited with a 405 nm continuous‐wave laser, and the emission was collected using a 100× objective lens (NA = 0.9, ZEISS). Time‐resolved fluorescence measurements were performed on an AXR‐NSPARC super‑resolution confocal fluorescence lifetime system (Nikon, Japan). Time‑correlated single photon counting (TCSPC) was employed for photon detection. The excitation source was a 405 nm pulsed laser with a repetition rate of 20 kHz, ensuring that the excitation pulse repetition period was sufficiently long to avoid pile‑up effects.

## Author Contributions

**Anjun Huang**: resources, supervision. **Zhengwen Yang**: resources, supervision. **Jingbo Sun**: supervision, resources. **Haonan Shi**: conceptualization, methodology, data curation, investigation, validation, formal analysis, visualization, writing – original draft, writing – review and editing. **Ningning Song**: investigation, writing – review and editing. **Yongzheng Wen**: supervision, resources. **Ji Zhou**: conceptualization, funding acquisition, project administration, resources, writing – review and editing, supervision.

## Conflicts of Interest

The authors declare no conflicts of interest.

## Supporting information




**Supporting File**: advs76954‐sup‐0001‐SuppMat.docx.

## Data Availability

The data that support the findings of this study are available from the corresponding author upon reasonable request.
