# Supplementary material for: Giant Magnetic Purcell Effect Induced by High‐Order Mie Resonances
Source: Adv Sci (Weinh). 2026 Aug 7;13(60):e76954. doi: 10.1002/advs.76954 (PMC13449752; doi:10.1002/advs.76954)
Supplement: Supplementary file 1 [file ADVS-13-e76954-s001.docx]

**Giant Magnetic Purcell Effect Induced by High-Order Mie Resonances**

Haonan Shi ^a^, Ningning Song ^a^, Anjun Huang ^b^, Zhengwen Yang ^b^, Jingbo Sun ^a^, Yongzheng Wen ^a^, Ji Zhou ^a#^

Figure S1. Magnetic field distributions of different Mie-resonant modes supported by a TiO_2_ sphere at the resonance wavelength of 590 nm under 1 V/m plane-wave excitation.

Figure S2. Radiative rate enhancement factors for magnetic and electric dipoles positioned 2 nm from the surface of a TiO_2_ sphere.

(a) Magnetic dipole orientated parallel to the surface; (b) Magnetic dipole orientated perpendicular to the surface; (c) Electric dipole orientated parallel to the surface; (d) Electric dipole orientated perpendicular to the surface.

Figure S3. Effect of material loss on the radiative rate enhancement factor of an isotropic emitter positioned 2 nm from the surface of a TiO_2_ sphere.

(a) *k*=0.0001; (b) *k*=0.001; (c) *k*=0.01; (d) *k*=0.1

Figure S4. Radiative rate enhancement factors for isotropic emitters near TiO_2_, Si, and Au spheres at a distance of 2 nm from the surface.

(a-b) TiO_2_ sphere with refractive index *n*=2.45 and *k*=0; (c-d) Si sphere: the refractive index values (*n*, *k*) are taken from literature ^[1]^; (e-f) Au sphere: the refractive index values (*n*, *k*) are taken from literature ^[2]^.

Figure S5. Effect of the emitter-TiO_2_ surface distance on the radiative rate enhancement factor of the magnetic dipole transition.

Figure S6. Effect of synthesis parameters on the morphology of TiO_2_ spheres.

(a-c) varied temperature at a fixed H_2_O/TTIP ratio of 8:1; (d-f) varied H_2_O/TTIP ratio at a fixed temperature of 0 °C.

Figure S7. SEM image and EDS mapping of TiO_2_–OH@Eu(TTA)_3_bpdc.

Figure S8. SEM image of P-TiO_2_–OH@Eu(TTA)_3_bpdc.

Figure S9. SEM image of TiO_2_–OH@Eu(TTA)_3_bpdc on a silica substrate.

(a) Numerical labels provide coarse identification of individual spheres. (b) The relative spatial arrangement of neighboring spheres enables precise localization of the target spheres.

Figure S10. Dark field image of TiO_2_–OH@Eu(TTA)_3_bpdc on a silica substrate.

Figure S11. Simulated radiative rate enhancement factors and relative branching ratios for the ^5^D_0_→^7^F*_j_* (*j*=0-4) transitions of Eu^3+^ ions.

(a) *j*=1 (magnetic dipole transition); (b) *j*=2 (electric dipole transition); (c) *j*=0 (electric dipole transition); (d) *j*=3 (electric dipole transition); (e) *j*=4 (electric dipole transition).

Figure S12. Photoluminescence spectra of P-TiO_2_–OH@Eu(TTA)_3_bpdc under 405 nm excitation.

Figure S13. Multipole decomposition of the scattering spectra presented in Figure 3(b–e).

(a) MQ, (b) MO, (c) M16-pole, (d) M32-pole.

Figure S14. Dark-field scattering and photoluminescence spectra of TiO_2_–OH@Eu(TTA)_3_bpdc spheres with resonance wavelength at ~580 nm.

Figure S15. Time-resolved fluorescence at 590 nm of P-TiO_2_–OH@Eu(TTA)_3_bpdc.

(The excitation source was a 405 nm pulsed laser with a repetition rate of 100 Hz, and multi-channel scaler (MCS) was employed for photon detection.)

**Reference**

[1]	M. A. Green, *Sol. Energ. Mater. Sol. C.* **2008**, 92, 1305.

[2]	A. D. Rakić, A. B. Djurišić, J. M. Elazar, M. L. Majewski, *Appl. Opt.* **1998**, 37, 5271.
